# A Micro Oxygen Sensor Based on a Nano Sol-Gel TiO_2_ Thin Film

**DOI:** 10.3390/s140916423

**Published:** 2014-09-03

**Authors:** Hairong Wang, Lei Chen, Jiaxin Wang, Quantao Sun, Yulong Zhao

**Affiliations:** State Key Laboratory for Manufacturing Systems Engineering, School of Mechanical Engineering, Xi'an Jiaotong University, Xi'an 710049, Shaanxi, China; E-Mails: chenlei1900621@gmail.com (L.C.); wangjiaxin666@126.com (J.W.); sqt871123@163.com (Q.S.); zhaoyulong@mail.xjtu.edu.cn (Y.Z.)

**Keywords:** O_2_ sensors, sol-gel, nanostructured, MEMS (Micro-Electro-Mechanical System), thin films, TiO_2_

## Abstract

An oxygen gas microsensor based on nanostructured sol-gel TiO_2_ thin films with a buried Pd layer was developed on a silicon substrate. The nanostructured titania thin films for O_2_ sensors were prepared by the sol-gel process and became anatase after heat treatment. A sandwich TiO_2_ square board with an area of 350 μm × 350 μm was defined by both wet etching and dry etching processes and the wet one was applied in the final process due to its advantages of easy control for the final structure. A pair of 150 nm Pt micro interdigitated electrodes with 50 nm Ti buffer layer was fabricated on the board by a lift-off process. The sensor chip was tested in a furnace with changing the O_2_ concentration from 1.0% to 20% by monitoring its electrical resistance. Results showed that after several testing cycles the sensor's output becomes stable, and its sensitivity is 0.054 with deviation 2.65 × 10^−4^ and hysteresis is 8.5%. Due to its simple fabrication process, the sensor has potential for application in environmental monitoring, where lower power consumption and small size are required.

## 1. Introduction

Traditional semiconductor gas sensors for relevant gases like CO, H_2_, NO_x_ and O_2_ based on metal oxides have been thoroughly investigated due to their good sensitivity, excellent chemical stability and reversible operation [1]. Titanium dioxide (TiO_2_) has attracted much attention of researchers since it is a typical metal oxide material which can work in harsh environments such as toxic atmospheres and bad temperature conditions. In the past decade, some reports on TiO_2_ thick-film sensors were published [2–5]. Meanwhile, attempts were also carried out to develop the TiO_2_ thin-film sensors. For example, Francioso developed TiO_2_ thin-film oxygen sensors to control the oxygen concentration in combustion processes in automobile engines in order to replace the traditional zirconium oxide oxygen sensors [6–10]. Thin-film metal oxide semiconductor sensors can be easily integrated with MEMS (Micro-Electro-Mechanical System) micro-hotplates, which results in lower power consumption compared to traditional ones based on thick-film processes.

The preparation of TiO_2_ thin films with good response properties is a precondition for developing a gas microsensor using such a kind of material. The properties of the films depend on the preparation process and doping method used. The sol-gel method can be used to deposit TiO_2_ thin films with nano-particles and high specific area. The sensing properties of pure TiO_2_ thin films are very weak and can be improved by adding doping materials such as Cr, Pt and Nb, *etc.* To fabricate microsensor-based TiO_2_ thin films, Francioso and Epifani used the dry etching process [7,10]. However, dry etching tends to change the properties of the deposited TiO_2_ films, with negative effects on the sol-gel films.

In this paper, a micro O_2_ sensor which consists of a nano sol-gel TiO_2_ thin-film board and a pair of interdigitated electrodes was investigated. A wet etching process was applied to define the TiO_2_ thin-film pattern, and after comparing the quality of the etched board with that fabricated by the dry etching process, wet etching was finally applied in the micro gas sensor fabrication. A pair of micro interdigitated electrodes was fabricated on the board by a lift-off process. X-ray diffraction analysis (XRD) and scanning electron microscopy (SEM) revealed the crystal information and surface topography. Experimental results indicated that the performance of the microsensor is sensitive to O_2_ with good repeatability. Since the sensor was fabricated on a silicon wafer by the standard micromachining process, it could become an individual device with low power consumption which can be integrated with a micro hotplate in the future and play a useful role in environmental monitoring.

## 2. Design and Fabrication

### 2.1. Structure Description

**Figure 1.**
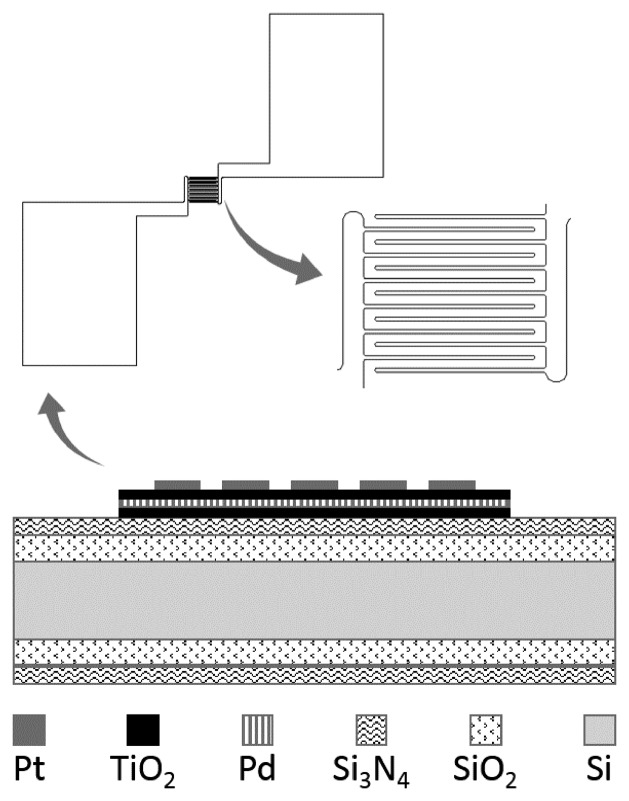
A schematic of the TiO_2_ oxygen sensor.

The schematic of the TiO_2_ oxygen sensor is illustrated in Figure 1. The TiO_2_ thin film structure was designed as a 350 μm × 350 μm square board, laid out on the surface of a silicon wafer with a deposited SiO_2_ and Si_3_N_4_ layer. On the surface of the TiO_2_ board, Pt electrodes were designed in an interdigitated pattern. With this design, one can easily fabricate Pt patterning and need not consider the possible influence of the subsequent micromachining process.

The TiO_2_ film consisted of three layers (TiO_2_/Pd/TiO_2_) in a sandwich structure, within which the buried Pd serves as the doping material. More details can be found in our previous work [11]. To simplify the micro gas sensor, in this paper no micro hotplate was integrated.

### 2.2. Fabrication Process

Fabrication of the micro O_2_ sensor was completed by a micromachining process, starting from a bare Si substrate. A SiO_2_ layer (the thickness of the SiO_2_ film was about 500 nm) was deposited by thermal oxidation and then a Si_3_N_4_ layer (150 nm) was deposited by low pressure chemical vapor deposition (LPCVD). To get the sandwich structure, a TiO_2_ film was formed by a sol-gel process first, and afterward a Pd thin film was deposited by sputtering equipment (Model Explorer 14, Denton Vacuum, Moorestown, NJ, USA). Finally another layer of TiO_2_ was deposited. Once the TiO_2_ pattern was defined, the Pt electrodes can be obtained by the lift-off process. During fabrication of the sensor, two steps are important but primary: one is preparation of the nano TiO_2_ thin film and another is definition of the pattern of the thin film. In the following, we describe them in detail.

Preparation and fabrication of nano TiO_2_ thin films on a silicon substrate have been reported in some references [12–14]. For a micro gas sensor, the thin film is expected to be a nanostructured material, such as nanoparticles, porous structures, or any other nano-scale form. For this purpose, the sol-gel method was often used to deposit the pure TiO_2_ thin films [15,16]. One may start by applying spin-coating precursor solution at 2000 rpm onto 4 inch (10.08 cm) oxidized silicon substrates for 30 s, and then the substrate is dried at 80 °C for 30 min and subsequently annealed at 500 °C for 3 h in ambient clean air. With this process, a first layer of TiO_2_ was successfully deposited with a thickness of 54 nm.

Then the buried Pd layer was deposited on the TiO_2_ thin film by DC (Direct Current) magnetron sputtering of a pure Pd target using the sputtering system as mentioned above, and then annealed at 500 °C for 2 h. The sputtering time was set to 10 s and its thickness is about 5 nm. Subsequently, the 2nd layer of pure TiO_2_ thin film, also with thickness of 54 nm, was deposited onto the buried Pd layer with the same process as that of the first one. By this process a sandwich TiO_2_/Pd/TiO_2_ thin film, namely, Pd doped TiO_2_ nanostructured thin film was prepared.

The next step was to define the pattern of the sandwich film. Both wet etching and dry etching were tried to fabricate the pattern. Lithographs for both processes were the same. A layer of positive photoresist (EPG 533) was spun onto the wafer, the rotation rate of the spinner was set to 1500 rpm and the resist thickness about 2 μm was prepared.

**Figure 2.**
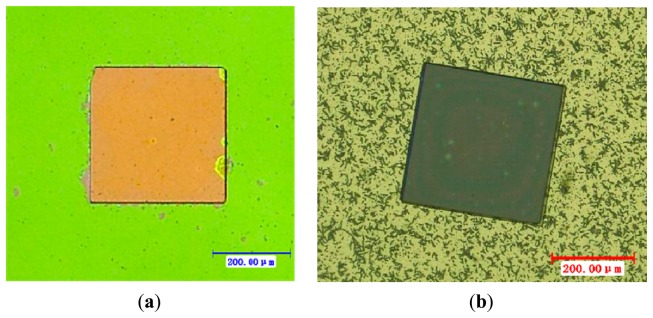
Optical microscope images of square board Pd buried nano TiO_2_ thin film. (**a**) Result by wet etching process; (**b**) Result by dry etching process.

When the wet etching process was used, the sample with the resist pattern was dipped into diluted hydrofluoric acid (HF and H_2_O with the volume ratio of 1:5). The reaction between HF and titania etched the TiO_2_ film. After 1 min, the sample's surface was wiped gently with swabs. Then the sample was placed into acetone and cleaned with an ultrasonic washer for 5 min, and thus a square board (350 μm × 350 μm) of thin TiO_2_ film was patterned on the sample, as shown in Figure 2a.

To find a more reasonable process, dry etching was also investigated to fabricate the square board (350 μm × 350 μm) of titania thin films. This was realized in an ICP (Inductively Coupled Plasma) system (ICP 180, Oxford Instruments, Oxford, UK). The heavy isotropic etching was oriented towards a 100 mTorr chamber pressure when a 40 sccm total flow of SF_6_, and a 1.5 W/cm^2^ power RF density were applied to the reactor wafer [7]. The highly controllable process allows a 2 μm resolution sensitive film patterning process. The process lasted for 6 min and the board of titania produced is shown in Figure 2b.

As shown in Figure 2a, the nonfunctional area had been etched more cleanly after wet etching and the square board nano TiO_2_ film was well shaped. However, for the etched board shown in Figure 2b, many irregular unevennesses may be seen, which may be TiO_2_ remaining on the nonfunctional area or perhaps the SiO_2_ layer and Si_3_N_4_ layer have been overetched and Si was exposed. In any case, one caneasily see that the wet etching produced a better result.

Subsequently, the micro interdigitated electrodes were defined by the UV lithography technology. The 150 nm Pt micro interdigitated electrodes with 50 nm Ti buffer layer were sputtered on the TiO_2_ thin film board by DC magnetron sputtering and ultrasonic stripping.

**Figure 3.**
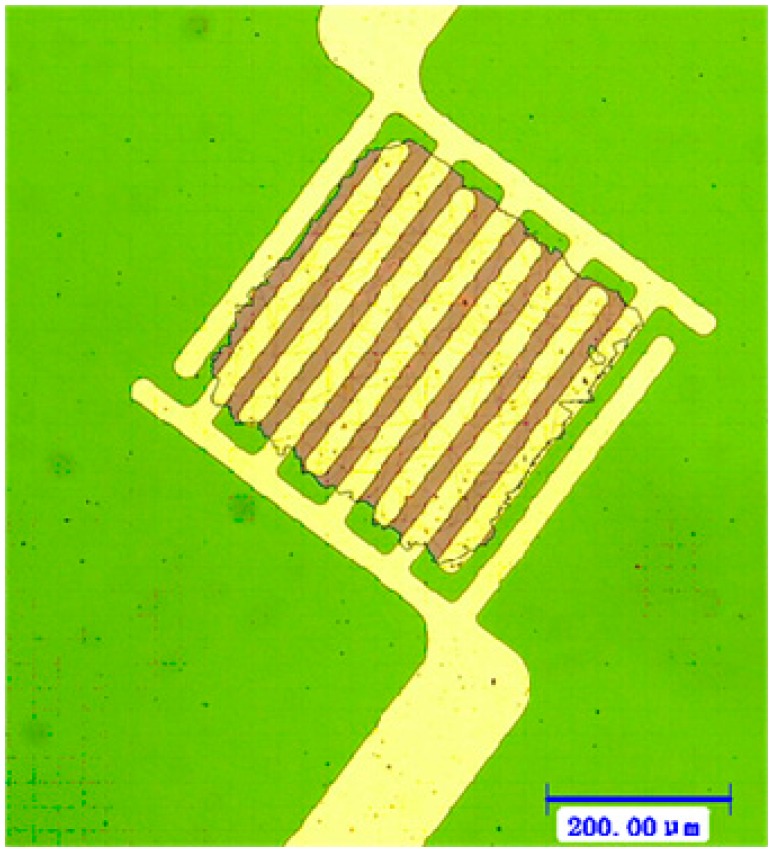
Optical microscope images of micro interdigitated electrodes on the sensitive film.

As shown in Figure 3, the Pt micro interdigitated electrodes were well-integrated with the sensitive film. The Ti buffer layer and the sensitive film have a better bonding than that of the platinum bonding with sensitive film directly. The photoresist lift-off process was operated gently to avoid fracturing the thin-film edge.

**Figure 4.**
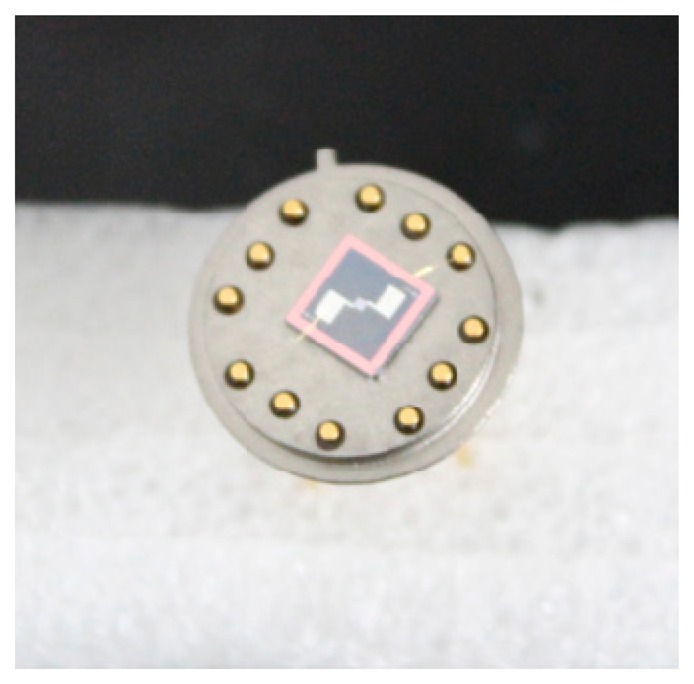
The mounted sensor chip.

A micro-machined silicon chip (5 mm × 5 mm) was glued on a TO-8 socket as shown in Figure 4. Golden wire bonding was used to make the electrical connections.

The crystalline phases of the pure TiO_2_ thin films and TiO_2_/Pd/TiO_2_ were characterized by X-ray diffraction (XRD, XRD-7000, Shimadzu, Tokyo, Japan) with Cu Kα radiation. Nanostructured pure TiO_2_ thin films and the doped TiO_2_ were observed by scanning electron microscope (SEM, Su-8010, Hitachi, Tokyo, Japan).

The electrical response of the samples recorded by a multimeter system (Agilent 34410A, Agilent Technologies, Santa, CA, USA) was carried out in a small chamber heated to 240 °C. The clean air was diluted by dry N_2_ of 99.999% purity and both gases were controlled with mass flow meters. Subsequently the diluted gas was led into the small chamber. The gas flow rate was kept at 200 sccm.

## 3. Results and Discussion

**Figure 5.**
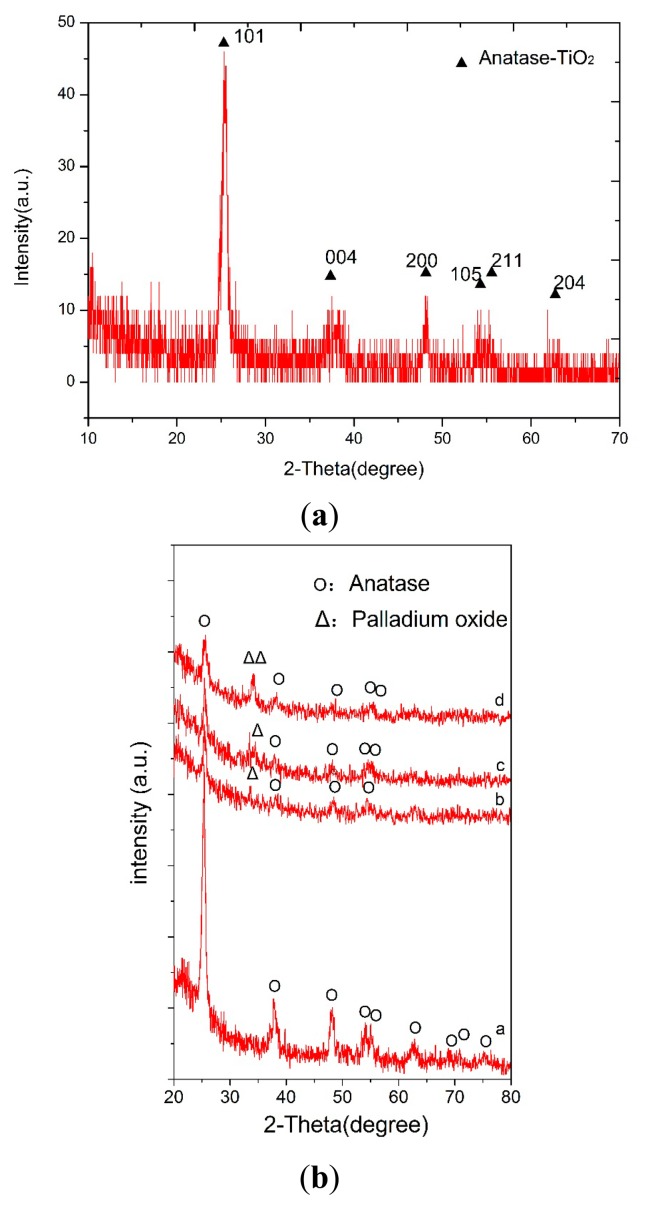
X-ray diffraction analysis (XRD) patterns. (**a**) XRD pattern of the pure TiO_2_; (**b**) XRD pattern of TiO_2_ annealed at 500 °C with different Pd sputtering times: a = 0.0, b = 5 s, c = 10.0 s, d = 20 s, reproduced with permission from [11].

**Figure 6.**
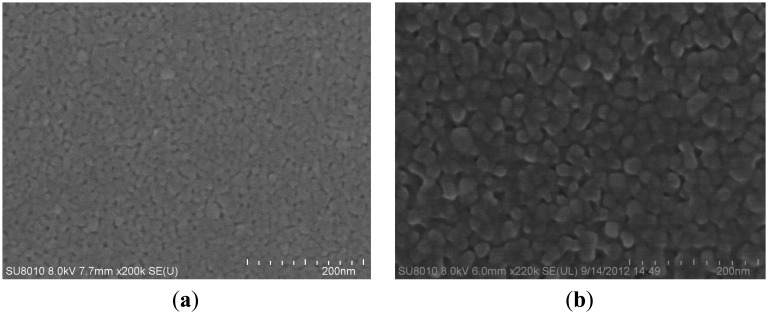
(**a**) SEM image of the TiO_2_/Pd/TiO_2_ film; (**b**) SEM image of the pure TiO_2_ thin film.

X-ray diffraction (XRD) spectra of the pure TiO_2_ thin films andTiO_2_/Pd/TiO_2_ thin films indicated that the phase of TiO_2_ is anatase, as shown in Figure 5a and Figure 5b, respectively. However, in the TiO_2_/Pd/TiO_2_ thin film, palladium oxide tends to appear, which could enhance the electrical conductivity at low temperature [17,18]. Anatase is usually the desired phase in gas-sensing devices [19,20]. Figure 6a is the SEM image of the TiO_2_/Pd/TiO_2_ film, which shows the sensitive film has a highly homogeneous and porous surface with nanoparticles. Compared with the pure TiO_2_ film as shown in Figure 6b, the TiO_2_/Pd/TiO_2_ film has smaller nanoparticle size, higher specific area, and smaller roughness. High homogeneity and small roughness are beneficial to design a micro sensor, since it is not necessary to consider placement and direction of the electrodes when we lay them out on the sensing film.

**Figure 7.**
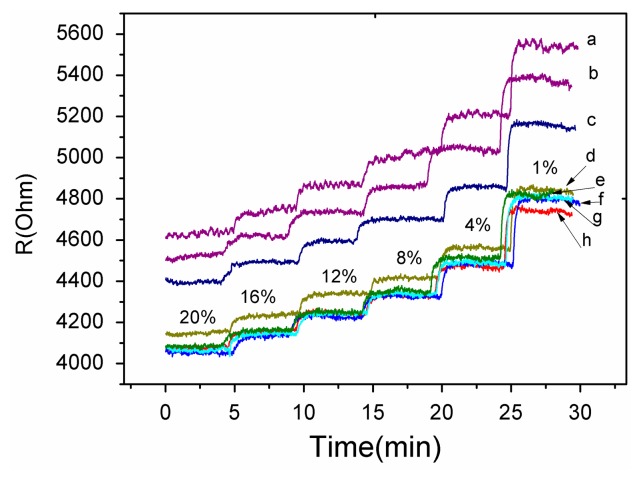
The response of the micro sensor based on TiO_2_/Pd/TiO_2_ thin film at 240 °C with different oxygen partial pressure. a, b, c, d, e, f, g, h curves were seven results of the chip in the order of testing cycles.

A gas sensing test was carried out to verify the response performance of the microsensor. The structure of TiO_2_/Pd/TiO_2_ thin films was tested with 1.0% to 20% oxygen partial pressure at 240 °C. Figure 7 shows the dynamic response and recovery time of the thin films. The sampling interval was set as 1 s and the raw data was plotted in Figure 7. The TiO_2_ thin films with Pd layer sputtered for 10 s showed good recovery at partial oxygen pressures ranging from 1.0% to 20%. The reproducibility of the different testing cycles of the sensor was tested by several testing process as shown in Figure 6. The test curves e, f, g and h tend to overlap, which indicates the diffusion of Pd has reached a steady state and the sensor based on the TiO_2_/Pd/TiO_2_ thin films can output a repeatable signal in different testing cycles.

The decrease of resistance with increasing concentration of O_2_ in Figure 7, which is indicative of p-type behavior, and the conductivity of the material is governed by holes [21]. However pure TiO_2_ thin films are a n-type semiconductor material. The diffused Pd layers provide acceptor impurities which can be expressed as:

(1) $$\text{PdO}↔Pd_{Ti}^{\prime \prime }+O_{o}+V_{o}^{••}$$

where ${\text{V}}_{\text{o}}^{••}$ denotes the oxygen vacancy and O_o_ represents oxygen atom. ${\text{Pd}}_{\text{Ti}}^{\prime \prime }$ is Pd substitution in Ti sites. The oxygen vacancies can react with oxygen andelectron holes will generate via Reaction (2) [16]:

(2) $$\frac{1}{2}O_{2}+V_{o}^{••}↔O_{o}+2{\text{h}}^{•}$$

These impurities make holes the dominant charge carriers in TiO_2_ thin films. Sinceholes act as the major carriers, O_2_ adsorption increases number of holes via Reaction (2), which leads to the decreasing resistance with increasing concentration of oxygen seen in Figure 7.

**Figure 8.**
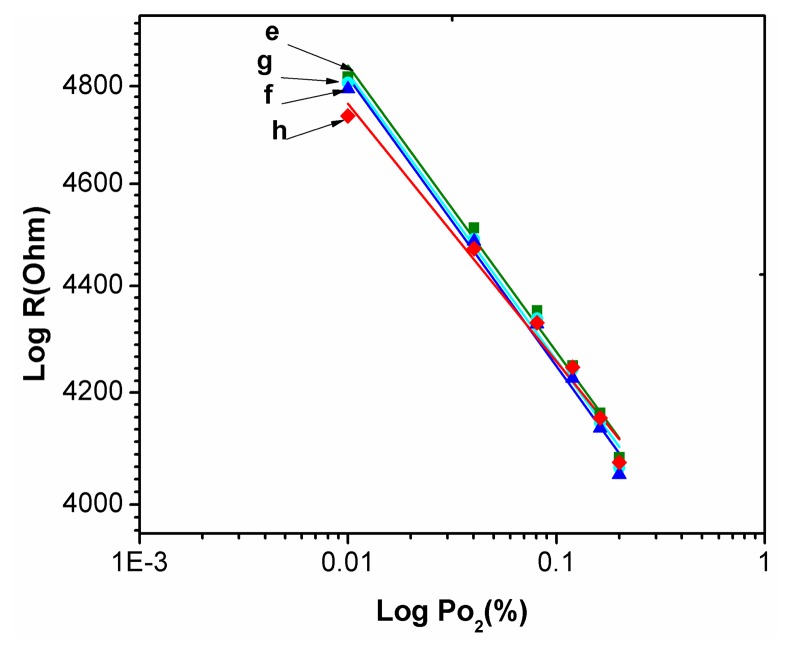
Relationship between the resistance R and the input oxygen partial pressure *Po*_2_.

Figure 8 showed the relationship between output resistance *R* and the input *Po*_2_ oxygen partial pressure for the static response of the TiO_2_ thin film with a buried Pd layer as *Po*_2_ varies from 1.0% to 20% at 240 °C. It is can be written as:

(3) $$R=R_{0}{\left(Po_{2}\right)}^{1/m}$$

where *R*_0_ is resistance of the film as *Po*_2_ zero, the *S* = *1/m* is defined as the sensitivity [15]. *R*_0_ can be obtained from the intercept of the fitting line. In Figure 8 the absolute values of *S* of the curves e, f, g, h are 0.05417, 0.05468, 0.0543 and 0.0487, respectively. The standard deviation of *S* was 2.65 × 10^−4^.

The Pd metal ion plays an important role in producing more active oxygen vacancy sites or the incoming oxygen molecules. The concentration of oxygen vacancies will increase as shown in Equation (1). Then the rate of recombination of oxygen vacancies with adsorbed oxygen molecules will be higher. The rate of appearance of holes is also higher. Then the result leads to a large change in the resistivity of the semiconducting oxide and higher sensitivity to oxygen. The fast movement of oxygen vacancies is responsible for a shorter response time [12,15].

**Figure 9.**
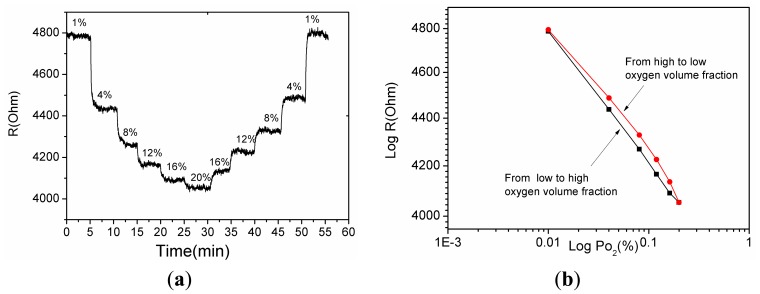
(**a**) The recovery response of the micro gas sensor; (**b**) The hysteresis characteristics of the micro gas sensor.

As shown in Figure 9a the sensor output was measured by gradually varying the oxygen partial pressure from 1% to 20% and again from 20% to 1%. Figure 9b shows that when increasing and decreasing the oxygen partial pressure the sensor output differs. The hysteresis is 8.5% and this probably arises from the different rates of adsorption and desorption.

**Figure 10.**
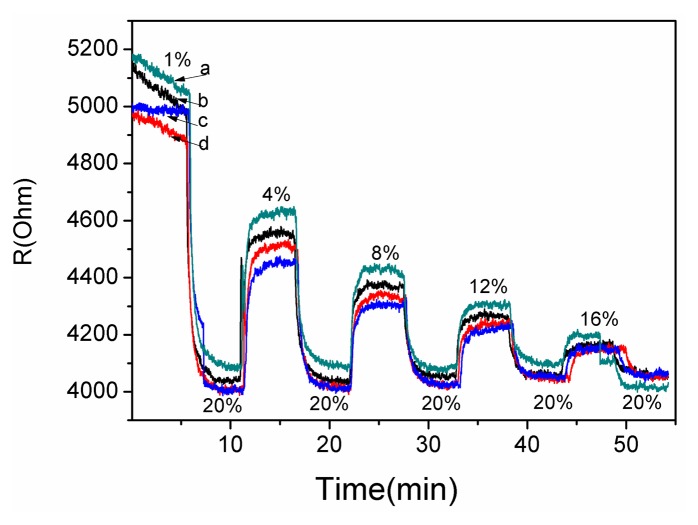
The response time of the gas microsensor at 240 °C with different oxygen partial pressures.

As shown in Figure 10, the average response time of curve d was approximately 52 s and the longest response time was 63 s. Here the response time was defined as the time required to reach 90% of the equilibrium readout. It was measured by switching the gas concentration from 20% and a certain concentration with a time interval 5 min. The micro size of sensitive thin films results in shorter response times than macro-size sensitive thin films [11], since at the micro scale the sensitive thin films can quickly approach gas adsorption equilibrium. The working temperature of the microsensor is 240 °C, which is lower than that reported by the recent literature [22], and the lower temperature is an obvious advantage for lowering the power consumption in real applications.

Selectivity is an important performance features for a gas sensor when there are some interfering gases in the atmosphere. CO_2_, SO_2_ and CO which are the main exhaust gases in the fossil fuel combustion process must be taken into account, and response properties of the sensor to these gases will be investigated in the future.

## 4. Conclusions/Outlook

An oxygen microsensor which uses a pattern of nano sol-gel TiO_2_ thin film with a buried Pd layer was presented. For the sensor microinterdigitated electrodes were laid out on the top of the TiO_2_ layer and with this design, the fabrication process was facilitated since wet etching can be applied to make the fabrication process simple and cheap. The response of TiO_2_ thin films with a buried Pd layer was observed at different oxygen pressures. The result indicates that the n-type of the TiO_2_ thin films was transformed to p-type behavior by inserting a buried Pd layer. After several cycles, the output signal becomes stable and repeatable. The sensitivity was 0.054 with a deviation of 2.65 × 10^−4^, the average response time was 52 s, and the hysteresis was 8.5%. Since the fabrication process is compatible with the MEMS process and it has exhibited good response properties, the proposed micro O_2_ sensor has potential for practical application. In the future we will focus on a method for integrating the Pd doped TiO_2_ thin films with a MEMS micro-hotplate to form an actual oxygen sensor.
